# Delirium among older adults living at home: a systematic review of prevalence, incidence, and risk factors

**DOI:** 10.3399/BJGP.2024.0456

**Published:** 2025-10-07

**Authors:** Karin E van Os, Marike C Schokker, Hendrika J Luijendijk

**Affiliations:** 1 Department of Primary and Long-term Care, University of Groningen, University Medical Centre Groningen, Groningen, the Netherlands

**Keywords:** delirium, epidemiology, older adults, primary health care, systematic review

## Abstract

**Background:**

Delirium will occur more often in community-dwelling adults as the population ages, increasing the a priori risk.

**Aim:**

To detearmine delirium prevalence and incidence among older adults living at home. The secondary aim was to examine delirium risk factors in this population.

**Design and setting:**

This was a systematic review guided by the Joanna Briggs Institute Manual for Evidence Synthesis.

**Method:**

PubMed/Medline, CINAHL, Embase, and Google Scholar were searched from inception to November 2024. Studies reporting delirium prevalence, incidence rate, or risk factors in individuals aged ≥65 years and residing at home were selected. Studies about delirium caused by alcohol or recreational drugs were excluded. Study characteristics, point prevalence, 1-year period prevalence or incidence, and risk factors were extracted and analysed.

**Results:**

Twenty-four studies were identified. The delirium prevalence rate was 0.2–8.3% in older community-dwelling adults, 1.4–44.1% in indviduals who were frail and received home care or who consulted a GP, and 16.0–27.0% in individuals referred for outpatient psychogeriatric care. The 1-year period prevalence and incidence was 0.0–5.8%. Studies that employed a delirium-specific instrument reported higher rates than studies based on routinely collected healthcare data. Twelve of the included studies examined 22 risk factors for delirium. A statistically significant association was found consistently with frailty, but not with demographics, medical history, or other factors.

**Conclusion:**

Delirium prevalence rates were substantial in community-dwelling older adults who were frail and people referred for outpatient psychogeriatric care. Studies relying on routine healthcare data showed the lowest prevalence rates, which suggests that delirium is often missed or not registered.

## How this fits in

It is generally assumed that delirium prevalence is low in community-dwelling older adults. However, this review found that studies have shown high delirium prevalence rates in community-dwelling older adults who are frail and who received home care or consulted a GP. Delirium is often present in people referred for outpatient psychogeriatric care. GPs may consider the use of a validated delirium instrument to support the detection of delirium in individuals with a high a priori risk.

## Introduction

Delirium is a serious geriatric syndrome commonly encountered in patients in hospital and long-term care residents.^1^ It is typically caused by underlying somatic illness or drug toxicity, both of which are often treatable. Following an episode of delirium, individuals face an increased risk of long-term cognitive impairment, psychological stress, prolonged hospital admissions, functional impairment, institutionalisation, and death.^2^

The prevalence of delirium in patients in hospital and long-term care populations is high, ranging from 25% to 40% in most studies.^3–6^ However, the prevalence of delirium has been studied less extensively in community settings. To the authors' knowledge, the first community-based study, which was published in 1991, reported a prevalence of 1% in a general population,^7^ reinforcing the assumption at the time that delirium prevalence was low among older people living at home. In contrast, a later study from 1998, found a much higher prevalence of 35% among community-dwelling older adults receiving home care.^8^

Delirium recognition and management in community-dwelling older adults presents unique challenges. It is widely known that delirium is difficult to recognise, with approximately half of cases missed in patients in hospital and long-term care populations. In community populations, recognition is further complicated by more subtle symptoms of delirium, and less apparent underlying somatic conditions. The absence of vigilant informal caregivers or continuous 24 h formal care can also impede recognition owing to the inherent fluctuation of delirium. Additionally, a lack of awareness of delirium in the general population may prevent patients and their families from seeking medical attention. Finally, missing delirium inadvertently confirms healthcare professionals in their assumption of a low a priori risk.

Previous research has identified various risk factors for delirium in hospital and long-term care facilities. In the latter, factors such as age, dementia, Parkinson’s disease, depression, malnutrition, urinary and respiratory infections, specific drugs (anticholinergics, antipsychotics, antidepressants, and benzodiazepines), and falls have been associated with an increased risk of delirium.^9^ However, a notable gap exists in the literature regarding risk factors specific to community-dwelling older adults. Understanding these risk factors is essential for improving delirium recognition within the community.

As the population ages, the prevalence of delirium among community-dwelling older adults is expected to rise.^10^ This underscores the need for a comprehensive understanding of delirium prevalence, specifically the a priori risk, to support GPs in identifying and managing new neuropsychiatric symptoms in a vulnerable group. The primary aim of this study was to determine the prevalence and incidence of delirium among older adults living at home. The secondary aim was to examine the risk factors for delirium in this population.

## Method

### Design

This systematic review followed the guidelines of the Joanna Briggs Institute (JBI) Manual for Evidence Synthesis^11^ and was reported in accordance with the PRISMA checklist. The protocol was registered in INPLASY (registration number INPLASY202410044).

### Search strategy and selection criteria

A comprehensive search of the following bibliographic databases for eligible studies was conducted: PubMed/Medline, CINAHL, Embase, and Google Scholar. Medical Subject Headings and keywords (or synonyms) were employed for the terms: delirium, older people, at home, risk factor and prevalence or incidence. The search string was initially developed in PubMed/Medline and then adapted for the other databases with the assistance of a librarian ( Supplementary Information S1). All hits yielded by PubMed/Medline, CINAHL, and Embase were screened and also the first 200 results in Google Scholar, as recommended.^12^ Backward and forward citation searching with each included article was also conducted. References were managed with EndNote 20. Searches were performed from inception to November 2024.

Two reviewers (the first and second author) independently screened titles and abstracts of the retrieved articles. Studies appearing to align with the eligibility criteria underwent full-text review for final inclusion. If a study appeared to collect relevant data not explicitly reported, the authors of the paper were contacted to request the data (Supplementary Information S2). Studies were only included if the necessary data were obtained. Reviewers compared and discussed their set of included studies until they reached agreement. If they could not reach agreement, the third reviewer was consulted.

Studies investigating delirium prevalence or incidence in community-dwelling adults aged ≥65 years were included, including cases in patients that may have originated during a recent hospital admission but persisted after discharge, as these individuals remain under their GP’s supervision on returning home. No limitations were applied regarding publication date or written language. Studies were excluded that focused on alcohol- or drug-induced delirium (recreational), those conducted in hospital or nursing home study populations (including emergency departments),^13,14^ studies in which delirium diagnoses were made solely in the hospital, and studies where it was not possible to isolate the target population from the data.

During the process of assessing full-text articles, a *post hoc* decision was made regarding the inclusion criteria, as the distinction between in- and outpatients was occasionally difficult to determine. Specifically, delirium diagnoses made in the emergency department were included if the text suggested that the patients remained under GP care.^15^ Furthermore, a decision was made to include two Dutch studies with approximately 16% care home residents^16,17^ and one Norwegian study with 25.2% of participants living in sheltered housing.^18^ In the Dutch context, a care home provides assisted living or light residential care under GP supervision. Similarly, people living in sheltered housing live independently in their own home, receive home care, and are under GP supervision. Finally, a study was included with 2.5% long-term care residents,^15^ because this small percentage is unlikely to have substantially influenced the reported delirium incidence. These populations were considered relevant for GP care, as these patients are under GP’s supervision (after returning home).

### Data extraction and risk of bias assessment

Two of three reviewers (the first author, paired with either the second author or senior author) independently extracted data from each included study. The following general study characteristics were extracted: country, setting, population, design, primary study aim, sample size, proportion female, dementia (percentage, all, some, or no participants), additional health issues affecting the overall population, and delirium assessment method (diagnostic or screening instrument, cutoff score, and diagnostic criteria). Additionally, point prevalence, period prevalence, and incidence, or the data to calculate these rates, were extracted including relevant statistical measures (such as standard errors and confidence intervals). Furthermore, data were extracted on any risk factors for delirium that had been studied and were found to be statistically associated with its occurrence.

Two of three reviewers (the first author, paired with either the second author or senior author) also assessed the methodological quality of the selected studies independently using the JBI critical appraisal checklist for prevalence studies.^11^ The JBI protocol does not provide specific cutoff values for determining the level of risk of bias, but these have been set for low and high risk in other reviews.^19,20^ In accordance with this practice, a risk of bias score of 8 or 9 was classified as low risk of bias (low risk of bias for all applicable items), 6 or 7 as medium risk (concerns in one or two applicable items), and <6 as high risk (concerns in at least three applicable items). The reviewers compared and discussed their data extractions and risk of bias assessments until they reached agreement. If they could not reach agreement, the third reviewer was consulted.

### Data synthesis

A figure was developed to display point prevalence for three subpopulations: the general community-dwelling older population, older people who were frail receiving home care or primary care, and older people receiving outpatient psychogeriatric care. In another figure, 1-year period prevalence and 1-year incidence for the same settings were combined, acknowledging the likely similarity between these measures given the transient nature of delirium. Where necessary, the 1-year period prevalence or incidence was computed, assuming a stable frequency over the year. In studies where prevalence or incidence was not reported but sufficient data were available (such as population size, study period, and number of people with delirium), the rates were calculated (Supplementary Information S3). Figures and calculations were produced using Stata SE version 18.0.

Clinical heterogeneity was assessed based on population characteristics (setting, age, dementia, and additional health issues affecting the overall population) and the screening or diagnostic method used. Owing to substantial heterogeneity in both study populations and methods, it was deemed unreasonable to pool the included studies in a meta-analysis. Alternative synthesis methods, such as Bayesian or thematic synthesis, were considered unsuitable as well, the former due to insufficient data on variability and precision, and the latter due to the lack of a predetermined conceptual framework. Therefore, a decision was taken to use descriptive statistics to summarise the results, organising these *post hoc* into three subpopulations.

In another *post hoc* decision, the risk factors were categorised in domains to account for variations in terminology across studies that examined similar factors. The risk factors found to be statistically associated with delirium are presented, as well as those that were not. However, the exact risk estimates are not reported owing to the variability in populations, methodologies, study quality, and risk measurement approaches across the included studies.

## Results

**Figure 1.**
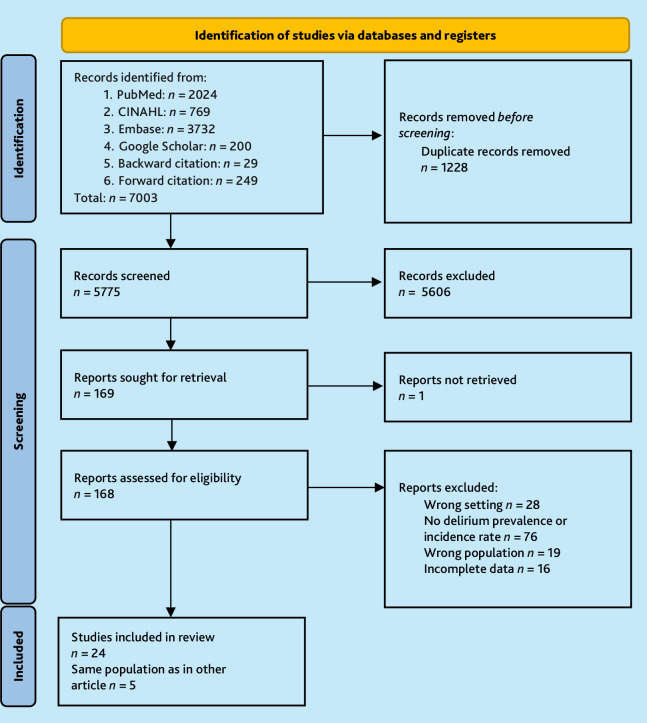
PRISMA flowchart for literature search

The search yielded 7003 records (see Figure 1 for PRISMA flowchart). In total, 168 full-text articles were assessed and 139 studies excluded for the following reasons: they did not pertain to community-dwelling populations or delirium prevalence or incidence; the data were incomplete relative to the research question; or the data specific to the population of interest could not be isolated from the study population.^21–25^

Twenty-six studies met the inclusion criteria. However, during the risk of bias assessment, it was noticed that two studies used an invalid assessment method.^25,26^ Specifically, one study defined possible delirium as a positive response to the question: ‘Do you have any of the following symptoms: confusion, disorientation, or drowsiness?’^26^ The other study defined delirium as the presence of an F20–F29 International Classification of Diseases (ICD) code^25^ for persistent delusional disorder. As a result, it was deemed inappropriate to include these studies.

Ultimately, 24 studies were included that were reported in 29 articles. If a population was studied more than once, the most relevant or comprehensive report was included. For example, the same population was described in a translated article,^27,28^ and the English article was included. Two articles reported on populations from the GErontological Regional DAtabase,^29,30^ and the larger cohort was included. In the excluded cohort of 393 individuals aged >85 years, the period prevalence was 7.9%. Four articles described overlapping (sub)populations,^31–34^ and the largest and most recent cohort was included in this review. The excluded cohorts comprised: 235 individuals aged >90 years, with a point prevalence of 26.0%;^33^ 116 individuals aged >60 years, with a diagnosis of Lewy bodies dementia and a point prevalence of 6.0%;^32^ and 615 individuals aged >65 years, with dementia and a point prevalence of 27.0%.^34^

The sample size across the studies ranged from 102 to 5 539 199 (Table 1). The research was conducted in Western Europe, North America, and Asia, and performed among:

- general community-dwelling older populations (*n* = 5);^7,29,35–37^
- older people who were frail receiving home care or primary care (*n* = 12);^8,15,18,27,38–45^ and
- older people receiving outpatient psychogeriatric care (*n* = 7).^16,17,31,46–49^

Outpatient psychogeriatric care involves care for people with cognitive impairment, with comprehensive medical examinations conducted in either the patient’s home^16,17,31^ (Dutch and Turkish studies) or at a clinic^49^ (Japanese study). The prevalence of delirium was based on diagnoses made by medical specialists.

One study included diagnoses from initial assessments in an emergency department, with 2.5% of its study population consisting of nursing home residents.^15^ Two Dutch studies conducted in outpatient psychogeriatric settings also included care home residents. A Norwegian study included sheltered housing residents.^16–18^

Four retrospective studies^15,27,39,42^ were based on delirium diagnoses derived from routinely collected healthcare data. Another study applied an algorithm to identify delirium symptoms in data of a cohort of older people that had been followed up to study dementia development.^41^ Eleven studies implemented an instrument to diagnose or screen for delirium prospectively.^8,16,18,29,31,37,40,42,44,45,49^ In total, 10 different sets of diagnostic criteria were used in the 24 included studies (Table 1).

Only five studies demonstrated a low risk of bias (a score of eight or nine).^16–18,47,49^ Fourteen studies did not use a validated method to identify delirium,^7,8,15,29,35–40,42,43,46,48^ and fourteen studies^7,15,27,31,37–43,45,46,48^ did not employ a standard, reliable way to measure delirium in all participants (Supplementary Table S1). Additionally, the sampling strategy was unclear or inadequate in five studies.^31,40,43,45,48^

**Table 1.** Characteristics of included studies

Study	Country	Population	Patients, *N*	Instrument^a^	Reference standard	RoB^b^	Risk factor^c^
**General community-dwelling older population**							
**Prevalence studies**							
Rabins (1996)^35^	US	Public housing residents, ≥65 years	865	No	DSM-III-R	7/9	–
Andrew (2006)^36^	Canada	No dementia, ≥65 years	1658	No	DSM-III-R	6/9	–
Folstein (1991)^7^	US	≥55 years	331	No	DSM-III	5/9	+
Vilalta-Franch (2009)^37^	Spain	≥70 years	602	No	DSM-IV	5/9	+
Eriksson (2011)^d,^ ^29^	Sweden/Finland	All female, ≥85 years	266	OBS	DSM-IV	6/9	–
**Older people who are frail receiving home care or primary care**							
**Prevalence studies**							
John (2014)^41^	Switzerland	Home care, 80% is ≥65 years	692	No	N/A	5/9	–
Vetrano (2016)^43^	Europe/Canada	Home care, ≥65 years	6903	RAI HC	Study specific	5/9	–
Hazra (2015)^42^	UK	Primary care, ≥100 years	11 084	No	N/A	6/9	–
Onder (2018)^40^	Europe	Home care, ≥65 years	1469	InterRAI HC	Study specific	4/9	–
Verloo (2016)^44^	Switzerland	Home care, discharged from hospital <48 h, ≥65 years	114	RAI HC	French CAM	6/9	–
Sandberg (1998)^8^	Sweden	Home care, ≥75 years	171	OBS	DSM-III-R	7/9	–
Tremolizzo (2021)^45^	Italy	Primary care, unable to visit GP, ≥65 years	102	4AT^e^	N/A	5/9	+
Bohlken (2018)^f,^ ^27^	Germany	≥1 visit to GP, ≥65 years	5 539 199	No	ICD-10 codes	5/9	–
Bowman (2020)^g,^ ^15^	UK	≥1 visit to GP, ≥65 years	85 887	No	N/A	6/9	+
Filderman (2024)^38^	Canada	Home care, ≥65 years	462 192	RAI HC	N/A	5/9	–
Delgado (2022)^39^	UK	Primary care, all dementia, ≥3 visit to GP, ≥65 years	9324	No	ICD-10 codes	6/9	+
Krogseth (2023)^18^	Norway	Home care, ≥65 years	210	Yes	DSM-V	8/9	+
**Older people receiving outpatient psychogeriatric care**							
**Prevalence studies**							
Stroomer-Van Wijk (2016)^17^	The Netherlands	Psychiatric referral, ≥55 years	275	DRS-R-98	DSM-IV	9/9	+
Hasegawa (2013)^49^	Japan	All dementia, ≥65 years	206	DRS-R-98	DSM-IV-TR	8/9	–
Quispel-Aggenbach (2021)^16^	The Netherlands	Referred for dementia screening, ≥65 years	373	DRS-R-98	DSM-IV-TR	9/9	–
Katipoglu (2023)^h,^ ^31^	Turkey	Tertiary outpatient clinic referral, all dementia, ≥65 years	721	Short CAM	DSM-IV/V	6/9	–
Holroyd (1997)^46^	US	Mood disorders treated with lithium, ≥65 years	114	No	N/A	5/9	–
Manni (2021)^47^	Switzerland	Memory clinic referral, all dementia, ≥65 years	2995	CAM	CAM	8/9	–
Lerner (1997)^48^	US	All dementia, ≥65 years	199	No	DSM-III-R	5/9	+

^a^An instrument designed to specifically diagnose, or screen, delirium used for the entire sample. No, if delirium diagnoses were based on routinely collected care data and registered clinical diagnoses. ^b^Risk of bias (RoB) score based on Joanna Briggs Institute Checklist for Prevalence Studies (part of the Critical Appraisal tools). ^c^Are risk factors studied in this study? + indicates that they were, and – that they were not. The investigated risk factors are presented in Figure 4. ^d^Mathilas *et al* ^30^ also studied a population within the same database, GErontological Regional DAtabase. ^e^The 4 A's Test for delirium (4AT) was used to screen, and the GPs then performed a clinical assessment in the screen-positive patients. ^f^The same studied population was described in a translated article.^28^ ^g^In total, 2.5% of the case group lived in a nursing home. ^h^Subpopulations were studied at earlier timepoints: 615 by Katipoglu *et al* (2022),^34^ 235 aged ≥90 years, by Veizi *et al *(2023),^33^ and 116 with Lewy bodies dementia aged ≥60 years by Naharci *et al* (2023).^32^ CAM = Confusion Assessment Method; DRS = Delirium Rating Scale; DSM = Diagnostic and Statistical Manual of Mental Disorders; DSM = Diagnostic and Statistical Manual of Mental Disorders; DSM = Diagnostic and Statistical Manual of Mental Disorders; N/A = Not Available; OBS = Organic Brain Scale; RAI HC = Resident Assessment Instrument Home Care; 4AT = 4 A’s Test for delirium

### Prevalence and incidence of delirium

**Figure 2.**
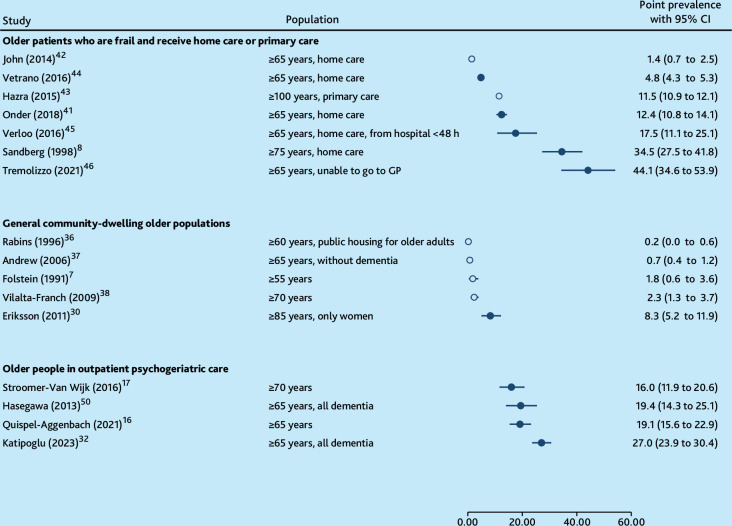
Point prevalence of delirium with older adults living at home. Clear dots (O) = no instrument designed to specifically diagnose or screen delirium used for the entire sample. CI = confidence interval.

Sixteen of 24 studies reported a point prevalence of delirium ranging from 0.2% to 44.1%^7,8,16,17,29,31,35–37,40–45,49^ (Figure 2). The lowest prevalence rates were reported in general community-dwelling older populations, ranging from 0.2% to 8.3%.^7,29,31,35–37^ In contrast, the highest point prevalence rates, along with the widest range, occurred among older people who were frail receiving home care or primary care, which ranged from 1.4% to 44.1%.^40–45^ In outpatient psychogeriatric care, point prevalence rates showed less variation, from 16.0% to 27.0%.^16,17,31,49^

Six of 16 studies did not use an instrument to screen for or diagnose delirium.^7,35–37,41,42^ Among these, five reported the five lowest point prevalence rates, ranging from 0.2% to 2.3%.^7,35–37,41^ The sixth study reported a point prevalence of 11.5% in a population of adults who were frail and aged >100 years receiving primary care.^42^

**Figure 3.**
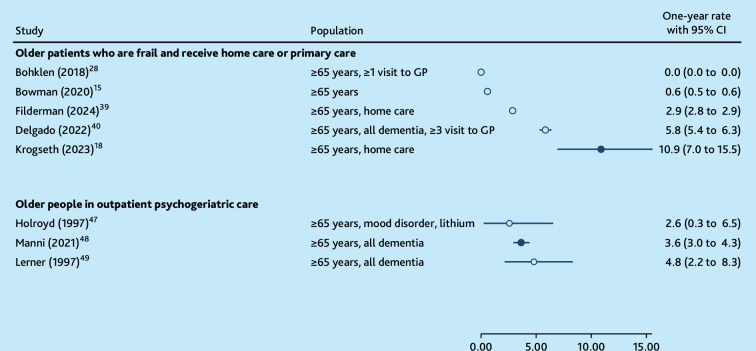
One-year period prevalence or 1-year incidence of delirium with older adults living at home. Clear dots (O) = no instrument designed to specifically diagnose or screen delirium used for the entire sample. CI = confidence interval.

Eight studies reported a 1-year period prevalence or incidence,^15,18,27,38,39,46–48^ which ranged from 0.0% to 10.9% (Figure 3). In older people who were frail and receiving primary care, the highest reported 1-year period prevalence was 10.9%.^18^ This prevalence was observed among older adults receiving home care at least once per week and who were monitored by the research team for delirium.^18^ A period prevalence of 0.0%, which corresponds to 0.006% when not rounded, was found in patients with at least one visit to the GP,^27^ while 0.6% was reported among all registered older patients.^15^

### Risk factors

**Figure 4.**
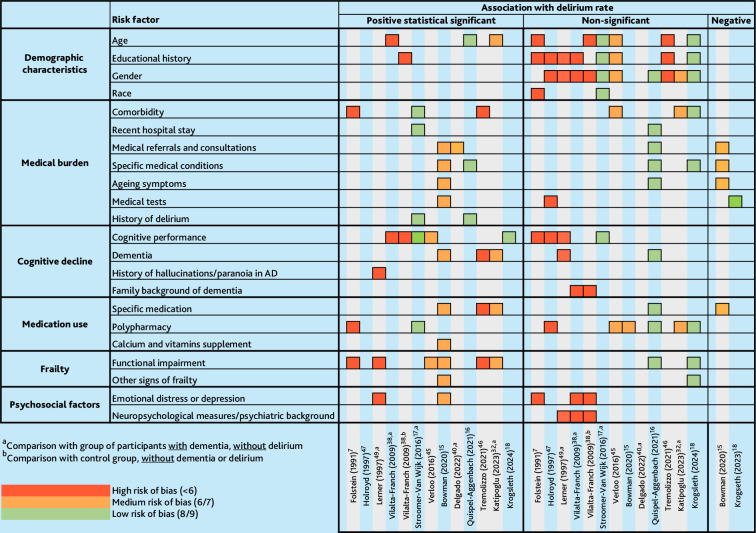
Risk factors investigated in 12 included studies. AD = Alzheimer’s disease.

Twelve of the 24 included studies examined 22 potential risk factors^7,15–18,31,37,39,44–46,48^ (Figure 4; Supplementary Table S2). The risk factors consisted of demographics (*n* = 10), medical burden (*n* = 11), cognitive decline (*n* = 11), medication use (*n* = 9), frailty (functional impairment) (*n* = 8), and psychosocial factors (*n* = 4). None of the studied demographic characteristics, including age, gender, educational level, and ethnic group, seemed to be consistently, statistically, or significantly related to delirium. Inconsistent results were found in the domains of medical burden, cognitive decline, medication use, and psychosocial factors. However, six of eight studies found a statistical association between the domain frailty and delirium. Notably, the studies in the review investigating risk factors did not exhibit a clear relationship between their findings and risk of bias.

## Discussion

### Summary

In this review, 24 studies were identified reporting heterogeneous delirium prevalence and incidence rates in older people living at home. Twelve of these studies examined 22 potential risk factors. Point prevalence ranged from 0.2% to 8.3% in general community-dwelling older populations, 1.4% to 44.1% in older people who were frail receiving home care or primary care, and 16.0% to 27.0% in outpatient psychogeriatric care. One-year period prevalence or incidence ranged from 0.0% to 10.9%.

Studies based on routinely collected healthcare data reported the lowest period prevalence and incidence rates in older people who are frail in primary care populations (0.0% to 5.8%). These lower rates may reflect underdiagnosis, likely because of the absence of instruments to diagnose or screen for delirium. In contrast, when one research team used an extensive study-specific method to diagnose delirium — including the Single Question in Delirium, an attention test, Richmond Agitation-Sedation Scale, and Observational Scale for Level of Arousal — the reported prevalence increased to 10.9%. Similarly, in general community-dwelling older populations, studies that did not use a diagnostic or screening instrument found the lowest point prevalence rates, ranging from 0.2% to 2.3%.^7,35–37^ When the Organic Brain Scale (OBS) was used, the reported point prevalence increased to 8.3%.^29^

Furthermore, if an instrument was used, the type of instrument seems to have influenced the reported delirium prevalence or incidence rates. For example, in three studies among older people who were frail receiving home care (and with no other additional health issues), the point prevalence was 4.8%, 12.4%, and 34.5%, respectively.^8,40,43^ The international Resident Assessment Instrument Home Care (interRAI HC) was used in the first two studies, whereas the OBS was employed in the third. The interRAI HC encompasses several items assessing cognitive, mood, and behaviour symptoms over the preceding 3 days. However, its focus on a limited timeframe and restricted delirium-specific symptoms may result in underdetection of certain people with delirium. In contrast, the OBS comprises numerous cognitive and neurological items, but lacks specific enquiry into acute onset. This approach may contribute to the overidentification of people with delirium. Although both instruments have been validated for other purposes, they are regarded as instruments for the identification of delirium.^50–52^

Across all subgroups, higher delirium prevalence rates were reported in frailer study populations. For example, the prevalence was 8.3% in women aged ≥85 years in general community-dwelling populations,^29^ 11.5% in adults aged ≥100 years in primary care,^42^ 27.0% in people with pre-existing dementia in outpatient psychogeriatric care,^31^ and 44.1% in people who were very frail and unable to go to the GP.^45^ Additionally, six of eight studies in the review that studied frailty as a risk factor for delirium showed a statistically significant association. However, these studies had a high risk of bias. The two studies that did not find a statistically significant association had a low risk of bias but relatively small sizes, potentially limiting the power to detect an effect.^16,18^ Nevertheless, their findings also suggest a possible association between frailty and delirium. No other demographic characteristic or other studied risk factors seemed to be consistently related with delirium.

### Strengths and limitations

To the authors’ knowledge, this is the first review of delirium prevalence and incidence in community settings. However, several limitations warrant further discussion. First, distinguishing between in- and outpatients is sometimes challenging. The review included initial diagnoses made in the emergency department,^15^ as well as four studies that had included small numbers of residents from sheltered housing, and care and nursing homes.^15–18^ Although the authors of the current study believe these populations are representative of community-dwelling adults and relevant to GPs, as outlined in the Method section, it is acknowledged that including these populations may have resulted in slightly overestimated delirium prevalence rates. Conversely, excluding admittance days in long-term care facilities was not possible in one study.^18^ Additionally, one study was included with a population aged ≥55 years,^7^ as it appeared to reasonably represent the older population. These two studies may have yielded somewhat underestimated prevalence rates.

Second, in calculating 1-year period prevalence, it was assumed that the follow-up periods of different studies reflected a calendar year. However, delirium prevalence may fluctuate seasonally, being potentially higher in hot summers (dehydration) and cold winters (infectious diseases).

Third, the methodological quality of the included studies likely affected the current findings. Over half of the studies exhibited a high risk of bias owing to the lack of a validated and standardised method of delirium identification. This may have led to underreporting. Additionally, an inverse relationship between large sample size and reported prevalence could reflect publication bias. However, the review did not assess this formally, as this is typically based on a pooled estimate.

Lastly, all but one study were conducted in high-income countries. Little is known about the prevalence, presentation, and aetiology of delirium in developing countries.^53^ This raises questions about generalisability.

### Comparison with existing literature

The highest prevalence rates of delirium were observed in studies using validated delirium instruments (3.6% to 44.1%), comparable with those observed in older people in nursing homes and hospitals.^3–6^ Notably, in three studies using the same validated diagnostic instrument (Delirium Rating Scale [DRS]-R-98) within a similar setting of psychogeriatric outpatient care, the reported point prevalence rates were remarkably consistent (16.0% to 19.4%). Therefore, caution is recommended when interpreting the relatively low prevalence rates reported in studies that did not use validated delirium instruments, as this may introduce a risk of bias.

Variations in prevalence may also stem from inconsistent diagnostic definitions and application. For example, even with the same diagnostic instrument applied in comparable populations, study-specific diagnostic criteria led to differing prevalence estimates from 4.8% to 12.4%.^41,43^ Likewise, even minor differences in diagnostic criteria (Diagnostic and Statistical Manual of Mental Disorders [DSM]-5, DSM-IV, Confusion Assessment Method [CAM], and DRS-R-98) resulted in identification of differing diagnosis of delirium in the same patients.^54^ One might expect that evolving diagnostic criteria, combined with an ageing population, a growing recognition of delirium as a distinct and serious condition, and an increasing prevalence of mental disorders, would yield higher diagnosis rates; however, this trend was not observed in this review. Similarly, reported estimates of delirium rates in medical inpatients have remained relatively stable over four decades.^3^

Frailty is suggested as a risk factor for delirium in hospitals and long-term care settings in other reviews as well as in this study.^3,5,55^ The current literature proposes that frailty predisposes to delirium, delirium triggers or accelerates frailty, or both conditions are manifestations of biological ageing.^55^ Frailty likely reflects a multidimensional, dynamic vulnerability, unlike static factors such as age. Nevertheless, although the association between frailty and delirium has been extensively studied,^55^ the pathophysiological pathways remain unclear. Similarly, most other potential risk factors, including comorbidity, recent hospital stays, specific medical conditions, and specific medications, have also been identified in patients in hospital and long-term care settings.^5,56^ Remarkably, none of the studies in this review reported the percentage of people with dementia in the study populations. Moreover, dementia was studied as a risk factor in only five studies,^15,16,31,45,48^ and history of delirium only in two,^16,17^ despite both being associated with delirium risk in other settings.^4,57–59^ Furthermore, inconclusive results were found concerning age, cognitive decline, and polypharmacy in the current study. Studies in long-term care and hospital settings typically show a statistically significant association between these factors and delirium.^5,56,60–62^ Perhaps this is an expression of the multifactorial aetiology of delirium, like that of frailty. Age or cognitive decline may only increase the risk of delirium when compounded by a serious precipitating factor, such as severe illness or surgery.

### Implications for research and practice

Delirium is likely underrecognised in older people living at home. Compared with patients in hospitals and long-term care settings,^63–66^ recognising delirium in community-dwelling patients is likely more challenging, as symptoms may be subtler, underlying illnesses less severe, and continuous (24 h) professional observation is often absent.

This review underscores the importance of heightened alertness for delirium in older adults who are frail and people that are referred to psychiatric services. The findings highlight a high a priori risk of delirium in these populations, which could enhance early recognition and improve patient care and outcomes in primary care settings. For example, earlier recognition could mean fewer medical crises in the future.

Delirium is likely best identified using a validated tool, both in research and clinical practice. The authors of the current study recommend screening high-risk populations with a validated instrument. For instance, an Italian study implemented a two-step procedure to identify delirium in older adults who were frail and unable to visit the GP.^45^ The GP first screened the patient with the <2-min 4 ‘A’s Test (4AT) and then diagnoses were made using conventional procedures in accordance with standard practice. Complementing psychiatric examinations with the DRS-R-98 also improved the recognition of delirium.^16^ Repeated use of such tools may eventually enhance professional recognition of delirium to the extent that the instrument becomes unnecessary. Future research should investigate how delirium can be better recognised in everyday general practice. Moreover, the risk factors for delirium in community-dwelling populations require further investigation to help GPs identify patients with an elevated a priori high risk. Additionally, future studies should aim to reduce risk of bias by using standardised, reliable, and valid methods for delirium identification across all participants.

## Data

The data used in this review can be found in the publication related to the included studies. The dataset that the authors compounded is available upon request from the corresponding author.

## References

[1] Wilson JE, Mart MF, Cunningham C (2020). Delirium. Nat Rev Dis Primers.

[2] Fong TG, Tulebaev SR, Inouye SK (2009). Delirium in elderly adults: diagnosis, prevention and treatment. Nat Rev Neurol.

[3] Gibb K, Seeley A, Quinn T (2020). The consistent burden in published estimates of delirium occurrence in medical inpatients over four decades: a systematic review and meta-analysis study. Age Ageing.

[4] Inouye SK, Westendorp RGJ, Saczynski JS (2014). Delirium in elderly people. Lancet.

[5] de Lange E, Verhaak PFM, van der Meer K (2013). Prevalence, presentation and prognosis of delirium in older people in the population, at home and in long term care: a review. Int J Geriatr Psychiatry.

[6] Koirala B, Hansen BR, Hosie A (2020). Delirium point prevalence studies in inpatient settings: a systematic review and meta-analysis. J Clin Nurs.

[7] Folstein MF, Bassett SS, Romanoski AJ, Nestadt G (1991). The epidemiology of delirium in the community: the Eastern Baltimore mental health survey. Int Psychogeriatr.

[8] Sandberg O, Gustafson Y, Brännström B, Bucht G (1998). Prevalence of dementia, delirium and psychiatric symptoms in various care settings for the elderly. Scand J Soc Med.

[9] Komici K, Guerra G, Addona F, Fantini C (2022). Delirium in nursing home residents: a narrative review. Healthcare (Basel).

[10] Hebert LE, Weuve J, Scherr PA, Evans DA (2013). Alzheimer disease in the United States (2010–2050) estimated using the 2010 census. Neurology (ECronicon).

[11] Aromataris E, Lockwood C, Porritt K (2020). JBI manual for evidence synthesis. https://jbi-global-wiki.refined.site/space/MANUAL.

[12] Haddaway NR, Collins AM, Coughlin D, Kirk S (2015). The role of Google Scholar in evidence reviews and its applicability to grey literature searching. PLOS ONE.

[13] Silva LOJE, Berning MJ, Stanich JA (2021). Risk factors for delirium in older adults in the emergency department: a systematic review and meta-analysis. Ann Emerg Med.

[14] Chen F, Liu L, Wang Y (2022). Delirium prevalence in geriatric emergency department patients: a systematic review and meta-analysis. Am J Emerg Med.

[15] Bowman K, Jones L, Masoli J (2020). Predicting incident delirium diagnoses using data from primary-care electronic health records. Age Ageing.

[16] Quispel‐Aggenbach DWP, Schep‐de Ruiter EPR, van Bergen W (2021). Prevalence and risk factors of delirium in psychogeriatric outpatients. Int J Geriat Psychiatry.

[17] Stroomer-van Wijk AJM, Jonker BW, Kok RM (2016). Detecting delirium in elderly outpatients with cognitive impairment. Int Psychogeriatr.

[18] Krogseth M, Davis D, Jackson TA (2023). Delirium, neurofilament light chain, and progressive cognitive impairment: analysis of a prospective Norwegian population-based cohort. Lancet Healthy Longev.

[19] Parola V, Coelho A, Cardoso D (2017). Prevalence of burnout in health professionals working in palliative care: a systematic review. JBI Database System Rev Implement Rep.

[20] Apóstolo J, Cooke R, Bobrowicz-Campos E (2018). Effectiveness of interventions to prevent pre-frailty and frailty progression in older adults: a systematic review. JBI Database System Rev Implement Rep.

[21] Chang CK (2013). Cognitive impairment and mortality in older clients of a secondary mental healthcare case register in London, UK. Eur Psychiatr.

[22] Davis DHJ, Muniz Terrera G, Keage H (2012). Delirium is a strong risk factor for dementia in the oldest-old: a population-based cohort study. Brain (Bacau).

[23] Lee SY, Wang J, Chao CT (2021). Frailty is associated with a higher risk of developing delirium and cognitive impairment among patients with diabetic kidney disease: a longitudinal population-based cohort study. Diabet Med.

[24] Lixouriotis C, Peritogiannis V (2011). Delirium in the primary care setting. Psychiatry Clin Neurosci.

[25] Wagenaar BH, Cumbe V, Raunig-Berhó M (2016). Outpatient mental health services in Mozambique: use and treatments. Psychiatr Serv.

[26] Zazzara MB, Penfold RS, Roberts AL (2021). Probable delirium is a presenting symptom of COVID-19 in frail, older adults: a cohort study of 322 hospitalised and 535 community-based older adults. Age Ageing.

[27] Bohlken J, Kostev K (2018). Prevalence and risk factors for delirium diagnosis in patients followed in general practices in Germany. Int Psychogeriatr.

[28] Bohlken J, Hewer W, Kostev K (2018). Incidence and treatment of non-substance-induced delirium in general and specialist practices in Germany. Fortschr Neurol Psychiatr.

[29] Eriksson I (2011.). Urinary tract infection: a serious health problem in old women. Umeå Universitet Medical Dissertations, New Series No 1410.

[30] Mathillas J, Olofsson B, Lövheim H, Gustafson Y (2013). Thirty-day prevalence of delirium among very old people: a population-based study of very old people living at home and in institutions. Arch Gerontol Geriatr.

[31] Katipoglu B, Demircan SK, Naharci MI (2023). Association of drug burden index with delirium in community-dwelling older adults with dementia: a longitudinal observational study. Int J Clin Pharm.

[32] Naharci MI, Kayahan Satis N, Ozsurekci C, Tasci I (2023). Assessment of clinical features and coexisting geriatric syndromes in newly diagnosed dementia with Lewy bodies: a retrospective study in a tertiary geriatrics setting in Turkey. Eur Geriatr Med.

[33] Veizi BGY, Taşcı İ, Naharci MI (2023). Geriatric syndromes in the population older than 90 years: the prevalence and association with chronic diseases. Australas J Ageing.

[34] Katipoglu B, Naharci MI (2022). Could neutrophil-to-lymphocyte ratio predict mortality in community-dwelling older people with delirium superimposed on dementia?. Aging Clin Exp Res.

[35] Rabins PV, Black B, German P (1996). The prevalence of psychiatric disorders in elderly residents of public housing. J Gerontol A Biol Sci Med Sci.

[36] Andrew MK, Freter SH, Rockwood K (2006). Prevalence and outcomes of delirium in community and non-acute care settings in people without dementia: a report from the Canadian study of health and aging. BMC Med.

[37] Vilalta-Franch J, Llinàs-Reglà J, López-Pousa S, Garre-Olmo J (2009). Prevalence and evolution of delirium in a community population of 70 years and older. Actas Esp Psiquiatr.

[38] Filderman B, Williams N, Mofina A, Guthrie DM (2024). What contributes to a decline in cognitive performance among home care clients? Analysis of interRAI data from across Canada. BMC Geriatr.

[39] Delgado J, Evans PH, Gray DP (2022). Continuity of GP care for patients with dementia: impact on prescribing and the health of patients. Br J Gen Pract.

[40] Onder G, Giovannini S, Sganga F (2018). Interactions between drugs and geriatric syndromes in nursing home and home care: results from shelter and IBenC projects. Aging Clin Exp Res.

[41] John G, Gerstel E, Jung M (2014). Urinary incontinence as a marker of higher mortality in patients receiving home care services. BJU Int.

[42] Hazra NC, Dregan A, Jackson S, Gulliford MC (2015). Differences in health at age 100 according to sex: population-based cohort study of centenarians using electronic health records. J Am Geriatr Soc.

[43] Vetrano DL, Foebel AD, Marengoni A (2016). Chronic diseases and geriatric syndromes: the different weight of comorbidity. Eur J Intern Med.

[44] Verloo H, Goulet C, Morin D, von Gunten A (2016). Association between frailty and delirium in older adult patients discharged from hospital. Clin Interv Aging.

[45] Tremolizzo L, Bargossi L, Storti B (2021). Delirium in your house: a survey during General Practitioner-programmed home visits. Aging Clin Exp Res.

[46] Holroyd S, Duryee JJ (1997). Differences in geriatric psychiatry outpatients with early- vs late-onset depression. Int J Geriatr Psychiatry.

[47] Manni B, Federzoni L, Zucchi P (2021). Prevalence and management of delirium in community dwelling older people with dementia referred to a memory clinic. Aging Clin Exp Res.

[48] Lerner AJ, Hedera P, Koss E (1997). Delirium in Alzheimer disease. Alzheimer Dis Assoc Disord.

[49] Hasegawa N, Hashimoto M, Yuuki S (2013). Prevalence of delirium among outpatients with dementia. Int Psychogeriatr.

[50] Björkelund KB, Larsson S, Gustafson L, Andersson E (2006). The organic brain syndrome (OBS) scale: a systematic review. Int J Geriatr Psychiatry.

[51] Salih SA, Paul S, Klein K (2012). Screening for delirium within the interRAI acute care assessment system. J Nutr Health Aging.

[52] Helfand BKI, D’Aquila ML, Tabloski P (2021). Detecting delirium: a systematic review of identification instruments for non-ICU settings. J Am Geriatr Soc.

[53] Paddick SM, Kalaria RN, Mukaetova-Ladinska EB (2015). The prevalence and clinical manifestations of delirium in sub-Saharan Africa: a systematic review with inferences. J Neurol Sci.

[54] Adamis D, Meagher D, Rooney S (2018). A comparison of outcomes according to different diagnostic systems for delirium (DSM-5, DSM-IV, CAM, and DRS-R98). Int Psychogeriatr.

[55] Bellelli G, Triolo F, Ferrara MC (2024). Delirium and frailty in older adults: clinical overlap and biological underpinnings. J Intern Med.

[56] Ahmed S, Leurent B, Sampson EL (2014). Risk factors for incident delirium among older people in acute hospital medical units: a systematic review and meta-analysis. Age Ageing.

[57] Bramley P, McArthur K, Blayney A, McCullagh I (2021). Risk factors for postoperative delirium: an umbrella review of systematic reviews. Int J Surg.

[58] Iglseder B, Frühwald T, Jagsch C (2022). Delirium in geriatric patients. Wien Med Wochenschr.

[59] Fong TG, Inouye SK (2022). The inter-relationship between delirium and dementia: the importance of delirium prevention. Nat Rev Neurol.

[60] Kubota K, Suzuki A, Ohde S (2018). Age is the most significantly associated risk factor with the development of delirium in patients hospitalized for more than five days in surgical wards: retrospective cohort study. Ann Surg.

[61] Hein C, Forgues A, Piau A (2014). Impact of polypharmacy on occurrence of delirium in elderly emergency patients. J Am Med Dir Assoc.

[62] Voyer P, Richard S, Doucet L, Carmichael PH (2009). Predisposing factors associated with delirium among demented long-term care residents. Clin Nurs Res.

[63] Wilson L, Power C, Owens R, Lawlor B (2019). Psychiatric consultation in the nursing home: reasons for referral and recognition of delirium. Ir J Psychol Med.

[64] Voyer P, Richard S, McCusker J (2012). Detection of delirium and its symptoms by nurses working in a long term care facility. J Am Med Dir Assoc.

[65] Eagles D, Fehlmann C, Emond M (2022). Why does delirium continue to go unrecognized?. CJEM.

[66] Ruangratsamee S, Assanasen J, Praditsuwan R, Srinonprasert V (2016). Unrecognized delirium is prevalent among older patients admitted to general medical wards and lead to higher mortality rate. J Med Assoc Thai.

